# Work-related adverse events leaving their mark: a cross-sectional study among Dutch gynecologists

**DOI:** 10.1186/s12888-018-1659-1

**Published:** 2018-03-22

**Authors:** Melanie A. M. Baas, Karel W. F. Scheepstra, Claire A. I. Stramrood, Ruth Evers, Lea M. Dijksman, Maria G. van Pampus

**Affiliations:** 1 Department of Obstetrics and Gynecology, OLVG, PO box 95500, 1090 HM Amsterdam, The Netherlands; 2 Department of Psychiatry, Academic Medical Center, PO Box 22660, 1100 DD Amsterdam, The Netherlands; 3 Department of Obstetrics and Gynecology, University Medical Center Utrecht, PO Box 85500, 3508GA Utrecht, The Netherlands; 4 Talmor, Andreas Bonnstraat 20hs, 1091AZ Amsterdam, The Netherlands; 5 Department of Research and Epidemiology, OLVG, PO box 95500, 1090 HM Amsterdam, The Netherlands; 6 Department of Research and Epidemiology, St. Antoniusziekenhuis, PO Box 2500, 3430EM Nieuwegein, The Netherlands

**Keywords:** Adverse events, Second victim, Obstetrician-gynecologist, Psychotrauma, Posttraumatic stress disorder, PTSD

## Abstract

**Background:**

Health care professionals who are frequently coping with traumatic events have an increased risk of developing a posttraumatic stress disorder. Research among physicians is scarce, and obstetrician-gynecologists may have a higher risk. Work-related traumatic events and posttraumatic stress disorder among obstetricians-gynecologists and the (desired) type of support were studied.

**Methods:**

A questionnaire was emailed to all members of the Dutch Society of Obstetrics and Gynaecology, which included residents, attending, retired and non-practicing obstetricians-gynecologists. The questionnaire included questions about personal experiences and opinions concerning support after work-related events, and a validated questionnaire for posttraumatic stress disorder.

**Results:**

The response rate was 42.8% with 683 questionnaires eligible for analysis. 12.6% of the respondents have experienced a work-related traumatic event, of which 11.8% met the criteria for current posttraumatic stress disorder. This revealed an estimated prevalence of 1.5% obstetricians-gynecologists with current posttraumatic stress disorder. 12% reported to have a support protocol or strategy in their hospital after adverse events. The most common strategies to cope with emotional events were: to seek support from colleagues, to seek support from family or friends, to discuss the case in a complication meeting or audit and to find distraction. 82% would prefer peer-support with direct colleagues after an adverse event.

**Conclusions:**

This survey implies that work-related events can be traumatic and subsequently can lead to posttraumatic stress disorder. There is a high prevalence rate of current posttraumatic stress disorder among obstetricians-gynecologists. Often there is no standardized support after adverse events. Most obstetrician-gynecologists prefer peer-support with direct colleagues after an adverse event. More awareness must be created during medical training and organized support must be implemented.

**Electronic supplementary material:**

The online version of this article (10.1186/s12888-018-1659-1) contains supplementary material, which is available to authorized users.

## Background

Professionals frequently coping with traumatic events have an increased risk of developing a posttraumatic stress disorder (PTSD). Groups at risk include military personnel, rescue workers, police, firefighters, and ambulance personnel. Similarly, hospital physicians cope with events such as severe illness, life-threatening situations and death frequently [1, 2]. However, research about traumatic events and PTSD among hospital physicians is scarce, and as a result the effects of work-related traumatic events may be underestimated. Overall, mental health problems in health care professionals may jeopardize the safety and quality of health care provided, with decreased productivity [3], high collateral costs [4], and medical errors [5–7]. Fortunately, the importance of physicians own mental health to foster optimal patient care is emphasized as a new competency in the CanMEDS framework [8]. The widely used CanMEDS framework was developed to define the necessary competencies for physicians. It provides a comprehensive foundation for medical education and practice for many (future) physicians in a dozen countries.. Obstetrician-Gynecologists (ObGyns) may be at increased risk of experiencing traumatic events since pregnancy and childbirth are expected to be joyful times, but can include severe complications, including stillbirth or maternal death with high emotional impact on the physician, midwife or nurse [9–13].

### Posttraumatic stress disorder

Whereas many types of work-related events can be marked as adverse, not all adverse events are traumatic. A traumatic event according to DSM-IV (Diagnostic and Statistical Manual of Mental Disorders) [14] is to experience or directly witness actual or threatened death, serious injury or a threat to the physical integrity of themselves or others. Furthermore, it was obligatory that the response involved intense fear, helplessness or horror. In the recently published fifth version of the DSM [15] this last criterion is removed, since it proved to have no utility in predicting the onset of PTSD. In particular, professionals did not always have these emotions at time of the event, while they did develop PTSD symptoms [16]. Furthermore, in the DSM-5, criterion A4 is added, specifically concerning professionals who have never been in direct danger, but can learn about consequences of traumatic events as part of their job [16].

Not all who experience a traumatic event develop PTSD: 80% of the general Dutch population will experience at least one traumatic event during their life, with a lifetime prevalence rate of PTSD being 7.4% [17]. A traumatic event can lead to a period of posttraumatic stress symptoms with intrusions, avoidance, negative cognitions and mood, and hyperarousal, but often these symptoms decline. When these symptoms last for at least one month, and lead to significant impairment in social, occupational or other important areas of functioning, PTSD can be diagnosed [15]. Risk factors for developing PTSD after experiencing a traumatic event include female gender, poor social support, prior trauma exposure, prior mental disorder and continuing stressors [18].

### Second victims

In 2000, the term second victim was introduced to address the impact of work-related adverse events on health care professionals [19]. Second victims can be defined as *“healthcare providers who are involved in an unanticipated adverse patient event, in a medical error and/or a patient related injury and become victimized in the sense that the provider is traumatized by the event”* [20]*.* The reported prevalence of second victims among health care providers varies from 10.4 to 46% [21–24]. The second victim can have continued emotional distress, leading to a posttraumatic stress disorder.

Studies about traumatic events and PTSD among health care professionals in general have low response rates and show a widely varying prevalence of PTSD or PTS-symptoms [1]. Studies among midwives, labor ward nurses and obstetricians reported a prevalence of moderate to severe work-related PTS-symptoms of 26–36%, with the most frequently reported traumatic events being fetal demise/neonatal death, shoulder dystocia (obstetric emergency during vaginal delivery that requires additional obstetric manoeuvres to deliver the fetus after the head has been born. It is associated with perinatal morbidity and mortality), and infant rescusitation [25–27].

### Support and coping after adverse events

It is unknown how many Dutch hospitals have implemented support strategies for health care professionals, but the absence of these strategies in many high risk specialties, such as anesthesiology is experienced as a common problem [28]. In addition, there is a low likelihood of a physician asking for help, due to perceived barriers (doubts about confidentiality, fear of negative impact on career, stigma [29], a low awareness of support options and time constraints [30].

More is known about the coping strategies of health care professionals after adverse events. The five most common coping mechanisms among American obstetricians are: asking support from a colleague, asking support from family or friends, exercising or performing hobbies, writing a formal case report or undertaking religious activities [10]. Other research supports this finding that physicians mainly wish for peer-support from colleagues after adverse events [30].

### Aim of the study

The aim of this study was to study the prevalence of work-related traumatic events (according to the DSM-IV A-criterion for posttraumatic stress disorder) among ObGyns, and to describe the prevalence of PTSD among ObGyns. Furthermore, we explored the current coping and professional support after work-related adverse events and the desired type of support. It was hypothesized that work-related events could be traumatic and lead to PTSD among ObGyns and current coping and support would be considered insufficient.

## Methods

### Population and procedure

The current study used the membership database of the Dutch Society of Obstetrics & Gynecology (NVOG). Permission to access and use this non-public database was granted by the NVOG. This database includes all resident and attending ObGyns in the Netherlands, as well as retired and non-practicing ObGyns. Of the 1596 members, 1578 members with a registered email address were included, which equals 98.9% of the NVOG population. Since the database did not differentiate between retired and non-practicing ObGyns, we considered all non-working ObGyns younger than 60 years into ObGyns with other jobs, and non-working ObGyns older than 60 years as retired. The link to the questionnaire was sent by email using an anonymous (non-traceable) link. All physicians received an invitation to participate in March 2014 by email, and two reminders during a 7.5 week period. The survey was piloted among a small group of resident and attending ObGyns for face validity, after which no substantial changes were made.

### Measurements

The survey consisted of 32 questions, starting with demographics (5 questions), personal experiences about work-related adverse events, coping and support provided (12 questions), desired support (2 questions) and one statement question. Lastly, when a work-related traumatic event was experienced during their career (PTSD-criterion A according to DSM-IV, 2 questions), at least once and more than four weeks ago, they also completed the Trauma Screening Questionnaire (TSQ) [31]. The TSQ is a validated 10-item screening instrument corresponding to a provisional diagnosis of PTSD according to the DSM-IV [14]. The Dutch psychometric properties are validated with Cronbach’s alphas from 0.71 to 0.91. A cut-off value of 6 or higher was used [32]. The original Dutch questionnaire and the English translation are included as Additional files 1 and 2 to this manuscript. The survey was part of a larger questionnaire among ObGyns about mental health after work-related adverse events.

### Statistical analysis

Descriptive and statistical analyses were performed with SPSS 18.0 for Windows. For the 4-point Likert scale questions, median scores for central tendency, interquartile range (IQR, 25–75%) and frequencies for distribution were calculated. Independents t-tests (for continuous variables) or Fisher’s exact tests (for categorical variables) were used to compare subgroups. All variables are reported with numbers (%) or mean ± standard deviation. A *p-*value of less than .05 was considered statistically significant. Open answers were independently categorized by two authors (KS and MB), and subsequently analyzed by two independent assessors(KS, MS). Any disagreement was discussed until consensus was achieved. The overall interrater reliability was moderate, with Cohen’s kappa’s of 0.44 or higher [33].

## Results

### Respondent and population characteristics

Table 1 shows the characteristics of the respondents (*n* = 683) and reference population. The sample was found to be a good representation of the NVOG members, as the response rates in the subgroups (age, gender, level of training) corresponded to the overall NVOG population. Of all the residents (*n* = 394), 184 (47.8%) responded, so the highest response rate was among residents, followed by attending ObGyns (45.7%). Retired and non-practicing ObGyns had a response rate of 25.3%. The majority of the respondents were female (65.3%), with a varying distribution among the subgroups. The percentages varied from 85% female residents to 14% female among retired ObGyns. This distribution corresponded to the tendency of having an increasing amount of young female medical doctors in the Netherlands. Work experience in obstetrics and gynecology ranged from 0.5 to 46.0 years. The respondent demographics are shown in Table 2.

**Table 1:** Demographic characteristics of respondents and NVOG population

Variable	Respondents *N* = 683	NVOG population *N* = 1596
ObGyn		
Resident	184 (26.9)	394 (24.7)
Attending	442 (64.7)	975 (61.1)
Non-practicing	21 (3.1)	18 (1.1)^a^
Retired	36 (5.3)	209 (13.1)^a^
Gender		
Male	237 (34.7)	663 (41.5)
Female	446 (65.3)	933 (58.5)
Age		
25–34	155 (22.7)	329 (21.2)
35–44	211 (30.9)	433 (27.9)
45–54	152 (22.3)	339 (21.8)
55–64	115 (16.8)	258 (16.6)
65 and older	50 (7.3)	194 (12.5)
Years in practice		
Mean ± SD	17.4 ± 10.7	Unknown
Range	0.5–46	Unknown
Complaints at disciplinary board	144 (21.1)	Unknown

All variables are in number (%), or mean ± standard deviation

*NVOG* Dutch Society of Obstetrics & Gynecology, *ObGyns* Obstetrician-gynecologists

^a^Calculated numbers. The NVOG did not differentiate between retired and not-practicing ObGyns. We counted ObGyns younger than 60 years as non-practicing, older than 60 years as retired

**Table 2:** Demographic variables per subgroup

	Total (*n* = 683)	Resident (*n* = 184)	Attending (*n* = 442)	Non-practicing (*n* = 21)	Retired (*n* = 36)
Gender					
Male	237 (34.7)	27 (14.7)	165 (37.3)	14 (66.7)	31 (86.1)
Female	446 (65.3)	157 (85.3)	277 (62.7)	7 (33.3)	5 (13.9)
Age					
25–34	155 (22.7)	146 (79.3)	9 (2.0)	0	0
35–44	211 (30.9)	38 (20.7)	169 (38.2)	1 (4.8)	3 (8.3)
45–54	152 (22.3)	0	149 (33.7)	3 (14.3)	0
55–64	115 (16.8)	0	107 (24.2)	6 (28.6)	2 (5.6)
65 and older	50 (7.3)	0	8 (1.8)	11 (52.4)	31 (86.1)
Years in practice					
Mean ± SD	17.4 ± 10.7	5.3 ± 2.4	20.6 ± 8.3	29.5 ± 9.3	32.4 ± 7.9
Complaints at the disciplinary board	144 (21.1)	3 (1.6)	118 (26.7)	8 (38.1)	15 (41.7)

All variables are in number (%), or mean ± standard deviation

### Posttraumatic stress disorder

The outcomes of the TSQ are shown in Table 3. 86 (12.6%) of the respondents reported having experienced at least one traumatic event during their work as an ObGyn and thereby met DSM-IV criterion A. One of them did not continue answering the TSQ, leading to 85 respondents completing the TSQ. 10 (11.8%) subjects screened positive for a current PTSD diagnosis. This equaled a prevalence of 1.5% among all ObGyns, and 1.4% among the ObGyns that are currently practicing (residents and attending). Among the remaining 75 ObGyns without current PTSD who did experience a traumatic event, 60.0% reported having experienced multiple work related PTS-symptoms earlier in their career. The sample size was not large enough to perform subgroup analysis on PTSD or PTS-symptoms earlier in life. The most commonly reported adverse events were neonatal death, maternal death, severe neonatal and maternal complications, patient aggression or violence towards healthcare professionals, medical errors and interpersonal conflicts with colleagues. Twenty-one respondents mentioned they did not wish to describe the traumatic event because of fear of loss of anonymity, three were not applicable (did not describe an event).

**Table 3:** Posttraumatic stress disorder measurements

	Total (*n* = 683)	Practicing (*n* = 626)		Not practicing (*n* = 57)	
		Resident (*n* = 184)	Attending (*n* = 442)	Other job (*n* = 21)	Retired (*n* = 36)
PTSD					
DSM-IV criterion A (%)	86 (12.6)	14 (7.6)	67 (15.2)	3 (14.3)	3 (8.3)
Above cut-off^a^ (%)	10 (1.5)	1 (7.1)	8 (12.1)	0 (0.0)	1 (33.3)

All variables are in number (%) of mean ± standard deviation

*DSM-IV* Diagnostic and Statistical Manual of Mental Disorders, *PTSD* Posttraumatic Stress Disorder, *TSQ* Trauma Screening Questionnaire

^a^Measured with a a TSQ cut-off value of 6 or higher. TSQ was completed by *n* = 85

### Work-related emotional stressors

Of all ObGyns, 230 (33.7%) have at some point considered leaving their medical profession. The most common reasons were a high workload, varying shifts, high responsibility, work/life imbalance, conflicts with colleagues, new interests and work culture related problems. 21.1% reported having faced a complaint at the disciplinary board, and this percentage increased with years in practice: 1.6% of the residents, 26.7% of the attending, 38.1% of the non-practicing and 41.7% of the retired ObGyns (Table 2). The group that had considered leaving the profession had a significantly higher PTSD prevalence rate (0.7% vs 3.0%, *p* = 0.01).

**Fig. 1:**
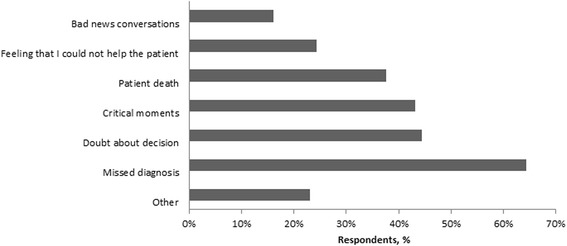
Events with high emotional impact

Respondents experienced the following events as high emotional impact stressors: missing a diagnosis (64.3%), doubting a medical decision (44.5%), life-threatening moments (43.2%), death of a patient (37.6%), feeling they could not help the patient (24.3%), bad news conversations (16.1%) (Fig. 1). Other stressors reported (23.1%) included severe complications, conflicts with colleague, patient and disciplinary board complaints, discontented patients and patient aggression or violence.

### Coping

**Fig. 2:**
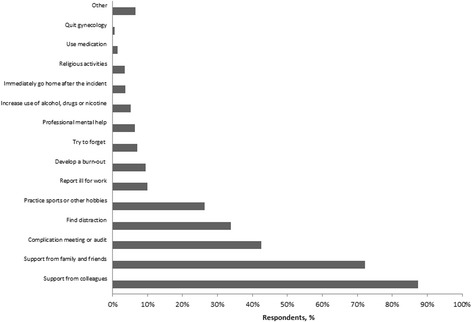
Coping strategies

The most commonly used coping strategies after emotional events (Fig. 2) were gaining support from colleagues (87.4%), gaining support from family or friends (72.2%), discussing the case in a complication meeting or audit (42.6%), finding distraction (33.8%) and practicing sports or other hobbies (26.4%). Among the respondents, 5.1% increased their use of alcohol, drugs and/or nicotine and 1.5% used medication they normally would not use. Furthermore, 0.6% gave up practicing as an ObGyn as a result of emotional stressors. When asked where they learned their coping strategies, 53% reported never having formally learned, 22.5% during a peer support group, 10.5% during specialist training, 4.8% during medical school, 4.8% during additional specialist courses and 33.8% learned through other ways. With the statement that there is room to express emotions on the ward or within their department after experiencing an emotional event, 80.8% agreed. Not many ObGyns (4.8%) agreed with the statement that having sleepless nights due to an adverse event means that you are not made to be an ObGyn. One in two ObGyns (55.2%) of the respondents have become more defensive in their decision making and 24.4% changed their work habits as a result of adverse events (e.g. no longer doing nightshifts, not performing surgery alone or no longer performing vaginal breech deliveries).

### Current and desired support

Of all ObGyns, 410 (60.0%) thought the current support services after adverse events are insufficient. Of these 410 respondents, 255 (62.3%) reported that their department or hospital has no support protocol or strategy, 105 (25.7%) were not aware whether there is a protocol and 49 (12.0%) do have a protocol. When asked about preferences for support after an adverse event, most ObGyns (82.0%) would prefer peer support from direct colleagues. 29.9% would like support from a professional (psychologist or counsellor), 22.3% would prefer peer support from indirect colleagues (non-ObGyn physicians) and 10% would like to have a buddy appointed. 86.1% stated that the culture concerning the support after adverse events should change.

## Discussion

In this large-scale study with a good response rate, our work-related PTSD prevalence among ObGyns (1.5%) is comparable to the general prevalence rate of current PTSD in the Netherlands (1.3%) [17]. However, this common prevalence is mostly not work-related, and with this in mind we conclude the work-related prevalence of PTSD among ObGyns is high compared to the general Dutch population. On the other hand, our estimated prevalence rate is low compared to other studies on prevalence rates of posttraumatic stress among health care professional in general (10.4–43.3%) [7, 21–24] and labor and delivery ward personnel (32–36%) [25–27]. However, a variety in definitions, questionnaires, stressor criterions, countries and professions is making comparison of results difficult. For instance, only one research group tried to determine a presumptive diagnosis of PTSD instead of posttraumatic stress in general, by applying the DSM-IV symptomcluster criteria to the Secondary Traumatic Stress Scale [25, 27]. It is needless to say that ObGyns who did not meet full criteria for PTSD may still have significant impairment, and it is important to look beyond a DSM-diagnosis.

Among the ObGyns without current PTSD who did experience a traumatic event, 59.2% report having experienced multiple work-related PTS-symptoms earlier in their career. Unfortunately, due to the retrospective aspect of this study, we cannot differentiate between physiological adaption, an Acute Stress Disorder or previous PTSD. Nonetheless, we conclude that psychological distress after a work-related adverse event is common.

While the CanMEDS framework is used during medical training in the Netherlands, the current study implies that coping and support are not learned during specialist training. Of the Dutch ObGyns, 60% think that the current support strategies are insufficient and only 12% reports that support services are available in their hospital/department*.* This corresponds to recent studies in which the institutional support for hospital-based physicians or nurses is considered low, and most support was to be expected from colleagues, family and friends [34–37]. With strong social support being an important protective factor for PTSD and gathering social support being a common coping mechanism in our sample, the role of educating health care professionals to support their colleagues after adverse events is crucial.

As a consequence of adverse events, ObGyns may become more defensive, adjust their work conditions and consider quitting their profession. One third of all Dutch ObGyns considered giving up gynecological practice, which is higher than findings from prior research (1 in 10) [10].^.^ However, in the present study the questions about considering giving up practicing did not differentiate between this being due to experiencing adverse events or because of other reasons. The results of this current study confirmed self-medication with (increased use of) drugs, nicotine and/or medication after experiencing an adverse event [23, 38] as a coping style used by some physicians after experiencing an adverse work-related event.

In accordance with prior research,^30^ situations described as high emotional impact are not necessarily the most extreme cases. Some physicians experience (multiple) minor complications as traumatic, whereas others experience disciplinary complaints or conflicts with colleagues as more stressful. We consider work conflicts and patient aggression or violence as work-related adverse events as well. These may concern all health care professionals and we therefore emphasize some form of support or guidance after such events as well.

### Strengths and limitations

One of the strengths of this study includes the large sample size (*n* = 683), as well as a high response rate of 42.8%. Although a recent study among Danish obstetricians and midwives about feelings and concerns after traumatic childbirth reached an even high response rate (59%) [39], our response rate is considerably higher than some previous studies (5–16%) [25–27]. There are also several limitations to this study. Since avoidance is inherent to PTSD and may lead to non-response, the PTSD-prevalence of 1.5% may be an underestimation. Due to the anonymous design of the study, a non-responder analysis was not possible. Secondly, PTSD criterion A was measured according to DSM-IV, in which experiencing intense fear, helplessness or horror right after the event was still required for a PTSD-diagnosis. In the DSM-5 this criterion has been removed, which is of particular interest since during work-related traumatic events there is often ‘the professional kicking in’, and emotions are postponed or neglected. This could have led to underdiagnosing traumatic events and thereby work-related PTSD. Another limitation is that a clinical interview is necessary for diagnosing PTSD, and therefore the prevalence rate in our study is an estimated prevalence.

### Implications and recommendations

For future research, we would advise to include questions about the time that has passed since the traumatic event, and to include possible confounders for developing PTSD after experiencing a traumatic event [40], which could help identifying and supporting high-risk individuals. If possible, a longitudinal design would be of great interest.

We suggest that all hospitals increase awareness among residents and attending physicians, and standardized support after adverse events should be implemented. Also, education on coping strategies should be expanded in medical training, to optimize peer support by colleagues.

Lastly, we suggest further evaluation of the most effective support methods and the effect on quality and safety of health care. We stimulate exploration of this topic among other medical specialties as well, since adverse events concern most health care professionals.

## Conclusion

This is the first large-scale study about work-related adverse events, coping, support and PTSD among Dutch ObGyns. Findings imply that there is a substantial group of ObGyns who experienced at least one work-related traumatic event (according to the DSM-IV criteria for PTSD), and that this can lead to work-related PTSD. As hypothesized, it was found that respondents consider the support after adverse events to be insufficient, and coping is not learned during medical and specialist training. There is potential for a change of culture, and creating a professional peer support system.

## Additional files

Additional file 1: Original Dutch version of the questionnaire. (PDF 133 kb)

Additional file 2: Questionnaire, translated in English. (PDF 52 kb)

## Abbreviations

DSM: Diagnostic and Statistical Manual of Mental Disorders
NVOG: Dutch Society of Obstetrics & Gynecology
ObGyns: Obstetrician-gynecologists
PTS: Post-Traumatic stress
PTSD: Post-Traumatic stress disorder
TSQ: Trauma screening questionnaire
