# Supplementary material for: Work-related adverse events leaving their mark: a cross-sectional study among Dutch gynecologists
Source: BMC Psychiatry. 2018 Mar 22;18:73. doi: 10.1186/s12888-018-1659-1 (PMC5863895; doi:10.1186/s12888-018-1659-1)
Supplement: Supplementary file 1 — Original Dutch version of the questionnaire. (PDF 133 kb) [file 12888_2018_1659_MOESM1_ESM.pdf]

Welkom bij de vragenlijst over de impact van belastende gebeurtenissen op de werkvloer.

Start de vragenlijst

Start

www.thesistools.com

1.

Geslacht*

- Vrouw
- Man

2.

Leeftijd*

- 25-34 jaar
- 35-44 jaar
- 45-54 jaar
- 55-64 jaar
- 65 jaar of ouder

3.

Do you need professional PDFs? Try **PDFmyURL!**

Huidige functie*

- AIOS
- Specialist / fellow
- Gynaecoloog in ruste
- Niet praktiserend / managementfunctie

4.

Aantal jaren werkzaam (geweest) binnen gynaecologie/obstetrie (inclusief werk als A(N)IOS)*

5.

Ik heb ooit overwogen te stoppen met het vak gynaecologie/obstetrie*

- Helemaal mee oneens
- Oneens
- Eens
- Helemaal mee eens

6.

Indien u ooit overwoog te stoppen met gynaecologie/obstetrie, wat was de belangrijkste reden daarvoor?*

- Belangrijkste reden:
- Nooit overwogen te stoppen

7.

Do you need professional PDFs? Try **PDFmyURL!**

Er is ooit een klacht tegen mij ingediend bij het tuchtcollege *

- ○ Nee
- ○ Ja

8.

De emotioneel meest belastende momenten van mijn werk vind ik (meerdere antwoorden mogelijk):

- ☐ Niet van toepassing
- ☐ Als ik slecht nieuws moet brengen
- ☐ De kritieke momenten waarop een patiënt in levensgevaar is
- ☐ Als een patiënt van me overlijdt
- ☐ Als ik een diagnose over het hoofd heb gezien
- ☐ Als ik het gevoel heb dat ik een patiënt niet kon helpen
- ☐ Als ik twijfel of ik wel de juiste beslissingen neem
- ☐ Anders, namelijk: ______

9.

Na het meemaken van de meest emotioneel belastende momenten die ik heb meegemaakt in de gynaecologie/obstetrie heb ik het volgende gedaan (meerdere antwoorden mogelijk):

- ☐ Niet van toepassing
- ☐ Professionele psychische hulp zoeken
- ☐ Zo snel mogelijk naar huis gaan
- ☐ Meer dan normaal alcohol, drugs of sigaretten gebruiken
- ☐ Medicatie gebruiken die ik normaal zelden/niet gebruik
- ☐ Geprobeerd er niet aan te denken
- ☐ Afleiding gezocht, proberen bezig te blijven
- ☐ Gebeden of mij tot andere religieuze activiteiten gewend
- ☐ Er een formele case review van gemaakt (complicatie-bespreking perinatale audit, SIRE procedure, melding IGZ)
- ☐ Gesport of mij beziggehouden met mijn hobby
- ☐ Erover gepraat met vrienden/familie

Do you need professional PDFs? Try **PDFmyURL!**

☐ Informeel erover gepraat met collega's
☐ Ik ontwikkelde klachten passend bij een burn-out (Bv: emotionele uitputting, depersonalisatie, verminderd zelfvertrouwen)
☐ Mij ziekgemeld
☐ Ik ben gestopt met verloskunde/gynaecologie
☐ Anders, namelijk: ______

10.

Ik vind de huidige vorm van opvang na belastende gebeurtenissen goed*

○ Helemaal mee oneens
○ Oneens
○ Eens
○ Helemaal mee eens

11.

Er is binnen mijn afdeling ruimte voor emoties na het meemaken van een emotioneel belastende gebeurtenis tijdens het werk*

○ Helemaal mee oneens
○ Oneens
○ Eens
○ Helemaal mee eens

12.

Het leren omgaan met emoties na emotioneel belastende gebeurtenissen heb ik (meerdere antwoorden mogelijk):

☐ Middels onderwijs tijdens mijn opleiding tot basisarts geleerd.
☐ Middels onderwijs tijdens mijn opleiding tot specialist geleerd.
☐ Middels geaccrediteerde cursussen/trainingen in het kader van mijn nascholing als specialist geleerd

Do you need professional PDFs? Try **PDFmyURL!**

- [ ] Bij intervisie geleerd
- [ ] Op een andere manier geleerd, namelijk: ____
- [ ] Nooit echt geleerd

13.

Ik heb naar aanleiding van patiëntgerelateerde nare gebeurtenissen mijn werkomstandigheden aangepast (bijv: geen diensten meer, geen vaginale stuitbevallingen meer, niet meer alleen opereren, meer/sneller in huis komen ipv. telefonische supervisie) *

- ( ) Helemaal mee oneens
- ( ) Oneens
- ( ) Eens
- ( ) Helemaal mee eens

14.

In de loop van mijn carrière ben ik defensiever geworden in mijn vakkundig handelen *

- ( ) Helemaal mee oneens
- ( ) Oneens
- ( ) Eens
- ( ) Helemaal mee eens

15.

Nadat ik een belastende gebeurtenis op de werkvloer had meegemaakt bestond de opvang uit: *

Do you need professional PDFs? Try **PDFmyURL!**

16.

Op mijn afdeling is er een protocol cq. vaste aanpak voor nazorg na belastende gebeurtenissen.*

- ◯ Nee
- ◯ Weet ik niet
- ◯ Ja, dit bestaat uit: ____

17.

Na het meemaken van een belastende gebeurtenis zou ik het liefst opvang krijgen in de vorm van:

- ☐ Evaluatie met directe collega's
- ☐ Intervisie met niet-directe collega's (geen gynaecologen, wel artsen)
- ☐ 1 op 1 gesprekken met een psycholoog of coach
- ☐ Een buddy
- ☐ Anders, namelijk ____

18.

Als je wakker ligt van heftige gebeurtenissen tijdens je werk ben je niet gemaakt voor dit vak*

- ◯ Helemaal mee oneens.
- ◯ Oneens
- ◯ Eens
- ◯ Helemaal mee eens

19.

Ik zou een cultuurverandering binnen de gynaecologie wat betreft de opvang na belastende gebeurtenissen stimuleren, ongeacht of ik het zelf nodig heb*

- ◯ Helemaal mee oneens

Do you need professional PDFs? Try **PDFmyURL!**
PDFmyURL

Oneens
Eens
Helemaal mee eens

Volgende pagina

Soms maken mensen zeer ingrijpende gebeurtenissen mee, zoals in een levensbedreigende situatie terecht komen door een ramp, een ernstig ongeluk of een brand; bedreigd of aangevallen worden; aangerand of verkracht worden; zien dat iemand vermoord wordt, of dood is, of ernstig gewond is; of horen dat er iets afschuwelijks is gebeurd met iemand die u na aan het hart ligt.

Als arts kan men ingrijpende patiëntenzorggerelateerde gebeurtenissen meemaken: (dreiging van) overlijden of ernstig letsel van een patiënt, maar ook bedreiging door een patiënt of familie.

20.

1) Heeft u ooit TIJDENS UW WERK ALS GYNAECOLOOG soortgelijke dingen meegemaakt?
2) Reageerde u bij tenminste één van deze gebeurtenissen met intense angst, hulpeloosheid of afschuw?
3) Heeft/hebben deze gebeurtenis(sen) minimaal 4 weken geleden plaatsgevonden?*

Ik heb alledrie de vragen met ja beantwoord
Nee, ik heb niet alledrie de vragen met ja beantwoord

Volgende pagina

Do you need professional PDFs? Try **PDFmyURL!**

Bekijk de onderstaande reacties die soms ontstaan na een traumatische gebeurtenis. Deze vragenlijst gaat over uw persoonlijke reactie op de traumatische gebeurtenis die u heeft meegemaakt. Geef aan (Ja / Nee) of u tenminste TWEEMAAL in de AFGELOPEN WEEK het volgende heeft ondervonden

NB Het gaat om uw persoonlijke reactie nu op de traumatische gebeurtenis.

21.

Verontrustende gedachten of herinneringen aan de gebeurtenis die in uw hoofd opkomen tegen uw wil in*

- Nee
- Ja

22.

Verontrustende dromen over de gebeurtenis*

- Nee
- Ja

23.

U gedragen of u voelen alsof de gebeurtenis opnieuw plaatsvindt*

- Nee
- Ja

24.

Do you need professional PDFs? Try **PDFmyURL!**

Van streek raken door dingen die u aan de gebeurtenis herinneren *

Nee
Ja

25.

Lichamelijke reacties (zoals snelle hartslag, steen in de maag, zweten, duizeligheid) wanneer u herinnerd wordt aan de gebeurtenis *

Nee
Ja

26.

Moeilijk in slaap kunnen komen of te vroeg wakker worden *

Nee
Ja

27.

Geïrriteerdheid of woedeaanvallen *

Nee
Ja

28.

Moeite hebben met concentreren *

Nee

Do you need professional PDFs? Try **PDFmyURL!**
PDFmyURL

Ja

29.

Verhoogd bewustzijn van mogelijke gevaren voor uzelf en anderen *

Nee
Ja

30.

Gespannen zijn of schrikken van iets onverwachts *

Nee
Ja

31.

Kunt u de situatie waarover u de bovenstaande vragen beantwoord heeft kort beschrijven?

32.

Het is mogelijk dat u bij bovenstaande vragen 'nee' invult wat betreft afgelopen week, maar dat u veel van bovenstaande symptomen in een EERDERE PERIODE in uw leven ervaren heeft na een gebeurtenis op uw werk. *

Nee, ik herken de symptomen niet

Do you need professional PDFs? Try **PDFmyURL!**

○ Ja, ik herken minstens 6 symptomen uit een eerdere periode in mijn leven

Volgende pagina

www.thesistools.com

Deze pagina dient ervoor om te weten te komen hoe u zich voelt. Lees iedere vraag goed door en geef uw antwoord aan met een kruisje in het hokje dat het beste weergeeft hoe u zich gedurende de afgelopen week gevoeld heeft.
Denk niet te lang na over een antwoord. Het gaat bij deze uitspraken om uw eigen indruk. Er bestaan geen foute antwoorden, elk antwoord is goed, zolang het maar uw eigen indruk weergeeft.

33.

Ik voel me gespannen *

○ Meestal
○ Vaak
○ Af en toe, soms
○ Helemaal niet

34.

Ik geniet nog steeds van de dingen waar ik vroeger van genoot *

○ Zeker zo veel
○ Wel wat minder
○ Duidelijk minder
○ Eigenlijk nauwelijks nog

Do you need professional PDFs? Try PDFmyURL!

35.

Ik heb een soort angstgevoel alsof er iets vreselijks zal gebeuren *

- Jazeker, en vrij erg
- Ja, maar niet zo erg
- Een beetje, maar het hindert me niet
- Helemaal niet

36.

Ik kan best lachen en de dingen van de vrolijke kant zien *

- Net zoveel als vroeger
- Nu wel wat minder
- Duidelijk minder
- Helemaal niet

37.

Ik maak me ongerust *

- Heel erg vaak
- Vaak
- Af en toe, maar niet zo vaak
- Heel soms

38.

Ik voel me opgewekt *

Do you need professional PDFs? Try **PDFmyURL!**
PDFmyURL

- Helemaal niet
- Heel af en toe
- Soms
- Meestal

39.

Ik kan best rustig zitten en me ontspannen*

- Jazeker
- Meestal
- Af en toe
- Helemaal niet

40.

Ik heb het gevoel dat alles moeizamer gaat*

- Bijna altijd
- Heel vaak
- Soms
- Helemaal niet

41.

Ik heb een soort angstig, gespannen gevoel in mijn buik*

- Helemaal niet
- Soms
- Vrij vaak
- Heel vaak

Do you need professional PDFs? Try **PDFmyURL!**

PDFmyURL

42.

Het interesseert me niet meer hoe ik eruit zie*

- Inderdaad, helemaal niet meer
- Niet meer zoveel als eigenlijk zou moeten
- Het interesseert me wel, maar iets minder dan vroeger
- Het interesseert me nog net zoveel als vroeger

43.

Ik ben onrustig en voel dat ik iets te doen moet hebben*

- Inderdaad, heel duidelijk
- Duidelijk
- Enigszins
- Helemaal niet

44.

Ik verheug me van tevoren op dingen die komen gaan*

- Net zoveel als vroeger
- Een beetje minder dan vroeger
- Veel minder dan vroeger
- Bijna nooit

45.

Ik raak plotseling in paniek*

- Inderdaad, zeer vaak
- Tamelijk vaak

Do you need professional PDFs? Try **PDFmyURL!**

- Soms
- Helemaal nooit

46.

Ik kan van een goed boek genieten, of van zoiets als een radio- of televisieprogramma*

- Vaak
- Tamelijk vaak
- Af en toe
- Zelden

Naar laatste pagina

www.thesistools.com

47.

Ruimte voor opmerkingen aangaande dit onderwerp:

Dank voor uw deelname!

Verstuur mijn antwoorden

Do you need professional PDFs? Try **PDFmyURL!**

Dank voor uw deelname!

Do you need professional PDFs? Try **PDFmyURL!**
