# Supplementary material for: Work-related adverse events leaving their mark: a cross-sectional study among Dutch gynecologists
Source: BMC Psychiatry. 2018 Mar 22;18:73. doi: 10.1186/s12888-018-1659-1 (PMC5863895; doi:10.1186/s12888-018-1659-1)
Supplement: Supplementary file 2 — Questionnaire, translated in English. (PDF 52 kb) [file 12888_2018_1659_MOESM2_ESM.pdf]

1. What is your gender?
    - Female
    - Male

2. What is your age?
    - 25-34 years
    - 35-44 years
    - 45-54 years
    - 55-64 years
    - ≥ 65 years

3. What is your current position?
    - Resident
    - Attending
    - Retired
    - Non-practicing / management

4. How many years have you been working in Gynecology/Obstetrics as a doctor? (including as intern)

    …………………………………………………

5. I have seriously considered quitting my job in Gynecology/Obstetrics
    - Strongly disagree
    - Disagree
    - Agree
    - Strongly agree

6. If applicable, what was the most important reason to consider quitting (more answers possible)?
    - Most important reason:
    - I never considered quitting

7. I have had a complaint against me at the disciplinary board
    - No
    - Yes

8. What work-related events do you consider the most emotional (more answers possibe)
    - Not applicable
    - Bad news conversation/interview
    - (Critically) ill patients
    - When a patient dies
    - When you miss a diagnosis
    - When I feel I can't help a patient
    - When I'm in doubt about whether you're making the right decision
    - Other…

9. How do you cope with the most emotional events on the work-floor (more answers possible)
    - Not applicable/ never experienced
    - Seeking professional help
    - Going home as soon as possible
    - Using (more) alcohol, drugs or cigarettes
    - Using new medication
    - Trying to not think about it

- Finding a distraction, staying active
- Praying or other religious activities
- Making it a formal case report (complication meeting, perinatal audit)
- Working out, enjoying my hobby
- Talking to friends and family
- Informally discussing the matter with peers/colleagues
- Developing burn-out symptoms (for example: emotional exhausted, depersonalisation, less confidence)
- Calling in sick
- Quitting my work in Gynecology/Obstetrics
- Other…

10. The current support organized by my institution after an adverse event is good:
    - Strongly disagree
    - Disagree
    - Agree
    - Strongly agree

11. There is plenty of room to informally discuss adverse events in the department/partnership:
    - Strongly disagree
    - Disagree
    - Agree
    - Strongly agree

12. Where did you learn to cope with adverse events? (more answers possible):
    - Med-school
    - Specialist-training
    - Training since I work as a ObsGyn
    - Intervision
    - Otherwise: ……………..
    - I have never learned to cope.

13. I have changed my work-conditions (e.g. less shifts, no vaginal breech deliveries, not operating alone anymore, staying in the hospital when on call) after experiencing a patient-related adverse event?
    - Strongly disagree
    - Disagree
    - Agree
    - Strongly agree

14. In the course of time of my carreer, I've become more defensive:
    - Strongly disagree
    - Disagree
    - Agree
    - Strongly agree

15. After a work-related adverse event, the support I received consisted of:

………………………………

16. There is a protocol available at my department regarding support after an adverse event:
    1. No
    2. I don't know
    3. Yes, it consists of……………..

17. My preferred support after an adverse event would be (more answers possible):
    - Evaluation with the present team
    - Intervision wit indirect collegaes (physicians but no gynecologists)
    - 1 on 1 conversation with psychologist or coach
    - A buddy system
    - Otherwise: ……………………

18. When you are having sleepless nights due to an adverse event means that you are not made to be an ObGyn.
    - Strongly disagree
    - Disagree
    - Agree
    - Strongly agree

19. There should be a change of culture regarding support after an adverse event:
    - Strongly disagree
    - Disagree
    - Agree
    - Strongly agree

Sometimes people experiences traumatic events, such as a live-theatening situation as a cause of a natural disaster, high-impact-trauma or fire; being attacked or raped; witness a murder, death of hear find out someone close to them experienced something terrible.
As a doctor, one can experience such events in patient-care: critical illness or death of a patient, severe injury, as well as violent behaviour from a patient or their family.

1) Have you ever, **<u>during your work as an ObGyn</u>**, experienced such(adverse) events/incidents?
2) Did you react with intense fear, helplessness or horror?
3) Did the event took place more than 4 weeks ago?

20.
    - My answer is yes to all three of the above questions
    - No, I did not answer all three of the above questions with "yes".

Read below about reactions than can develop after an traumatic event. This questionnaire is abut your personal reaction at the traumatic event. Answer (yes/no) if you have experienced <u>at leat twice</u> <u>in the past week</u> the following:

21. Upsetting thoughts or memories about the event that have come into your mind against your will
    - Yes
    - No

22. Upsetting dreams about the event
    - Yes
    - No

23. Acting or feeling as though the event were happening again
    - Yes
    - No

24. Feeling upset by reminders of the event
    - Yes
    - No

25. Bodily reactions (such as fast heartbeat, stomach churning, sweatiness, dizziness) when reminded of the event
    - Yes
    - No

26. Difficulty falling or staying asleep
    - Yes
    - No

27. Irritability or outbursts of anger
    - Yes
    - No

28. Difficulty concentrating
    - Yes
    - No

29. Heightened awareness of potential dangers to yourself and others
    - Yes
    - No

30. Being jumpy or being startled at something unexpected
    - Yes
    - No

31. Can you describe the event/incident?
    ………………………..

32. It is possible that you didn’t experience the reactions, such as described on the previous pages, over the past few weeks, but do recognize them from a previous time of your life after experiencing a work-related event

- No, I don’t recognize the symptoms
- Yes, I do recognize having at least 6 symptoms from a previous period in my life

---

The aim of this page is t find out how you feel. Please read every question carefully and check the box that applies the best how you’ve felt for the previous week.
Don’t think too long about it. In these questions, your own impression is most important. There are no wrong answers, and every answer is correct if it fits your own impression.

33. I feel tense or wound up.
    - Most of the time
    - A lot of the time
    - From time to time
    - Not at all

34. I still enjoy the things I used to enjoy.
    - Definitely as much
    - Not quite so much
    - Only a little
    - Hardly at all

35. I get a sort of frightened feeling as if something awful is about to happen.
    - Very definitely and quite badly
    - Yes, but not too badly
    - little, but it doesn’t worry me
    - Not at all

36. I can laugh and see the funny side of things.
    - As much as I always could
    - Not quite as much now
    - Definitely not so much now
    - Not at all

37. Worrying thoughts go through my mind.
    - A great deal of the time
    - A lot of the time
    - From time to time but not too often
    - Only occasionally

38. I feel cheerful.
    - Not at all
    - Not often
    - Sometimes
    - Most of the time

39. I can sit at ease and feel relaxed.
    - Definitely
    - Usually
    - Not often

- Not at all

40. I feel as if I am slowed down.
    - Nearly all the time
    - Very often
    - Sometimes
    - Not at all

41. I get a sort of frightened feeling like ‘butterflies’ in the stomach.
    - Not at all
    - Occasionally
    - Quite often
    - Very often

42. I have lost interest in my appearance.
    - Definitely
    - I don’t take as much care as I should
    - I may not take quite as much care
    - I take just as much care as ever

43. I feel restless as if I have to be on the move.
    - Very much indeed
    - Quite a lot
    - Not very much
    - Not at all

44. I look forward with enjoyment to things
    - As much as I ever did
    - Rather less than I used to
    - Definitely less than I used to
    - Hardly at all

45. I get sudden feelings of panic
    - Very often indeed
    - Quite often
    - Not very often
    - Not at all

46. I can enjoy a good book or TV program
    - Often
    - Sometimes
    - Not often
    - Very seldom

47. If you want, you can leave a comments about this topic:
    ………………………..
